# Comprehensive NGS profiling to enable detection of *ALK* gene rearrangements and *MET* amplifications in non-small cell lung cancer

**DOI:** 10.3389/fonc.2023.1225646

**Published:** 2023-10-20

**Authors:** Sergi Clavé, Jennifer B. Jackson, Marta Salido, Jacob Kames, Kelly M. R. Gerding, Ellen L. Verner, Eric F. Kong, Elizabeth Weingartner, Joan Gibert, Max Hardy-Werbin, Pedro Rocha, Xènia Riera, Erica Torres, James Hernandez, Gustavo Cerqueira, Donna Nichol, John Simmons, Álvaro Taus, Lara Pijuan, Beatriz Bellosillo, Edurne Arriola

**Affiliations:** ^1^ Pathology Department, Hospital del Mar, Barcelona, Spain; ^2^ Cancer Research Program, Hospital del Mar Medical Research Institute, Barcelona, Spain; ^3^ Centro de Investigación Biomédica en Red de Cáncer (CIBERONC), Madrid, Spain; ^4^ Personal Genome Diagnostics (PGDx/Labcorp), Baltimore, MD, United States; ^5^ Medical Oncology Department, Hospital del Mar, Barcelona, Spain

**Keywords:** next generation sequencing (NGS), non-small cell lung cancer (NSCLC), *MET* amplification, *ALK* rearrangement, fluorescence *in situ* hybridization (FISH), biomarkers, molecular testing

## Abstract

**Introduction:**

Next-generation sequencing (NGS) is currently widely used for biomarker studies and molecular profiling to identify concurrent alterations that can lead to the better characterization of a tumor’s molecular landscape. However, further evaluation of technical aspects related to the detection of gene rearrangements and copy number alterations is warranted.

**Methods:**

There were 12 *ALK* rearrangement-positive tumor specimens from patients with non-small cell lung cancer (NSCLC) previously detected via fluorescence *in situ* hybridization (FISH), immunohistochemistry (IHC), and an RNA-based NGS assay, and 26 *MET* high gene copy number (GCN) cases detected by FISH, selected for this retrospective study. All 38 pre-characterized cases were reassessed utilizing the PGDx™ elio™ tissue complete assay, a 505 gene targeted NGS panel, to evaluate concordance with these conventional diagnostic techniques.

**Results:**

The detection of *ALK* rearrangements using the DNA-based NGS assay demonstrated excellent sensitivity with the added benefit of characterizing gene fusion partners and genomic breakpoints. *MET* copy number alterations were also detected; however, some discordances were observed likely attributed to differences in algorithm, reporting thresholds and gene copy number state. TMB was also assessed by the assay and correlated to the presence of NSCLC driver alterations and was found to be significantly lower in cases with NGS-confirmed canonical driver mutations compared with those without (p=0.0019).

**Discussion:**

Overall, this study validates NGS as an accurate approach for detecting structural variants while also highlighting the need for further optimization to enable harmonization across methodologies for amplifications.

## Introduction

Lung cancer is one of the leading causes of cancer-related deaths in the world. (1) In the current era of precision medicine, non-small cell lung cancer (NSCLC) has a wide assortment of targeted therapies linked to genomic biomarkers and signatures. (2, 3) Progress in genomic analysis using next-generation sequencing (NGS) has enabled the comprehensive detection of multiple targetable alterations simultaneously from limited tumor tissue. (4) Despite this, single-variant and -gene approaches are still employed as standard procedure in many settings to detect clinically relevant driver mutations resulting in tissue exhaustion and, potentially, incomplete tumor characterization. Apparent discrepancies between NGS and these conventional approaches, particularly regarding gene rearrangements and amplifications, however, make the interpretation of some of these alterations by NGS technologies challenging (5–8).

*ALK* gene fusions are important predictive biomarkers for targeted tyrosine kinase inhibitor (TKI) efficacy in NSCLC (9, 10). *ALK* rearrangements were initially assessed by fluorescent *in situ* hybridization (FISH); however, the approach is laborious, costly, and highly subjective (5, 6, 11). Additionally, because this methodology relies on the detection of merged or discrete signals from fluorescent probes, certain *ALK* gene fusions may be undetected or false-positive patterns could also be reported (12). Increasingly, immunohistochemistry (IHC) has been proposed as an improved and simplified alternative to FISH in the detection of *ALK* fusions (11, 13). However, both techniques are cell-based, microscopic approaches, making the process inherently subjective, with additional uncertainty arising from borderline or inconclusive cases. Moreover, these approaches are heavily affected by tumor heterogeneity, depending on the tissue sections used for analysis, particularly due to the scarce material usually obtained for lung cancer diagnosis. Consequently, FISH or IHC can be discordant and inconclusive for *ALK* fusions (6, 7, 14).

*MET* amplification represents a therapeutic target in NSCLC, though the clinical consensus and standardization of its definition are still subject to controversy (15, 16). Currently, TKIs of MET, such as crizotinib initially, and more recently capmatinib and tepotinib, have demonstrated clinical efficacy and safety in lung cancer patients with *MET* exon 14 skipping mutations (17, 18) and *MET* gene amplification (19). Due to the availability of targeted therapies, molecular testing of *MET* is now included in routine clinical workup in the NSCLC setting with comprehensive NGS panels including analysis for both exon 14 skipping mutations and *MET* amplifications (20). Reasons for equivocal outcomes for *MET* amplification may be attributed to a lack of clinically defined cutoffs for *MET* copy number or fold change (21–23). When using FISH, *MET* amplifications can be measured by comparing the ratio of *MET* copies to chromosome enumeration probe 7 (CEP7) (19, 23, 24). FISH also enables differentiation of focal amplifications and polysomy; however, the clinical significance for each of these patterns remains unclear. Regarding NGS, the assessment of *MET* amplification depends on the platform and remains to be validated in a broad clinical context including associations with response to MET inhibitors (25, 26).

The comprehensive nature of NGS has also facilitated the creation of novel cancer signatures, previously not possible with conventional techniques, such as tumor mutation burden (TMB) (20). TMB acts as a composite genomic score, often reported as mutations per exome or mutations per megabase (muts/Mb) of DNA sequenced, which highlights the overall mutational load of a tumor (27, 28). Clinical observations in NSCLC show that TMB-high (TMB-H) tumors are more responsive to immune checkpoint inhibition (29, 30). This observation has been confirmed in other solid tumor types and has led to FDA approval of pembrolizumab in TMB-H cases, making TMB a new pan-solid tumor biomarker for immunotherapy (31).

Given the multitude of clinically relevant genomic biomarkers present in NSCLC, we sought to analytically evaluate the level of concordance between conventional molecular pathology approaches and a DNA-based NGS assay. Furthermore, we aimed to elucidate any associations between concomitant driver mutations, co-alterations, and explore correlations to clinical outcomes in specific cases.

## Materials and methods

As a referral testing center for lung cancer biomarkers, Hospital del Mar has tested over 3,950 NSCLC samples from 2012 to 2017 for *ALK*, *ROS1*, and *MET* alterations (32, 33). Samples that presented sufficient residual material after diagnosis were deposited in the biobank collection of our Institution (*MARBiobanc* integrated in the *Xarxa de Bancs de Tumors de Catalunya*). Only initial diagnostic samples with remaining FFPE tissue from NSCLC patients were selected for this study. There were 12 samples with known *ALK* fusion events previously identified by FISH (Abbott Molecular, Des Plaines, IL, USA) reviewed to determine the tumor area and screened by IHC (D5F3, Ventana, Tucson, AZ, USA) and RNA-based NGS (Oncomine™ Focus Assay, Thermo Fisher Scientific, Waltham, MA, USA). Furthermore, 26 advanced NSCLC cases screened for *MET* amplification by FISH using the Cappuzzo score system (*MET* high gene copy number (GCN) defined as those with a mean of ≥5 copies per cell) (24) were recategorized into the currently accepted FISH criteria as the following: high-amplification (*MET*/CEP7 ratio ≥4.0), medium-amplification (*MET*/CEP7 ratio >2.2–<4.0), low-amplification (*MET*/CEP7 ratio ≥1.8–≤2.2), and non-amplification (*MET*/CEP7 ratio <1.8) (19) (**Supplementary Figure 1**). Using these FISH criteria, NSCLC samples considered positive for the presence of *MET* amplification are those with a *MET*/CEP7 >2.2 (high- and medium-amplification), classifying low-amplified together with non-amplified as negative as no actionability was seen when using MET inhibitors (19). This project was approved by the local ethics committee (CEIC-PSMAR: 2015/6336/I), and all patients provided written informed consent.

All 38 samples were further evaluated by the Personal Genome Diagnostics (PGDx™) elio™ tissue complete, a research use only (RUO) comprehensive genomic profiling NGS assay (PGDx, Baltimore, MD, USA). This NGS panel assesses 505 genes using a minimum starting input of 50 ng (100 ng recommended) DNA from FFPE tumor tissue and identifies key genomic alterations (single-nucleotide variants (SNVs), insertion and deletions (indels), gene rearrangements, and amplifications) and complex genomic signatures including TMB, reported as muts/Mb (exome equivalent), and microsatellite instability status. *ERBB2* amplifications were called when >2.5-fold change on normalized read depth was detected, and all other gene amplifications represented on the panel were called when >3-fold change was observed. Quality control metrics evaluated by PGDx elio tissue complete are shown in **Supplementary Table 1**. Sample processing from FFPE tissue, including library preparation, hybrid capture, sequencing, and analysis was performed at PGDx. DNA from FFPE was extracted using the QIAamp® DNA FFPE Tissue Kit (QIAGEN, Hilden, Germany) according to the manufacturer’s protocol. Captured libraries were sequenced on the NextSeq® platform (Illumina, San Diego, CA, USA). Somatic variant identification was performed using the PGDx elio tissue complete bioinformatics software, which incorporates a machine learning-based variant calling algorithm to differentiate between true somatic mutations and artifacts or false positive signal (34). The resultant NGS calls were compared with available data from the orthogonal approaches and with patient clinical data. TMB and microsatellite status were also assessed by the PGDx elio tissue complete assay using previously described methods (35). Finally, cases with driver alterations were analyzed for concomitant aberrations and correlated with clinical features. The Mann–Whitney U test was used for continuous parameters. Statistical tests were conducted at the two-sided 0.05 alpha level of significance, carried out with SPSS Statistics software (SPSS Inc., Chicago, IL, USA).

## Results

### Clinical cohort overview

There were 38 specimens from patients with NSCLC utilized in this study, composed of 27 males and 11 females (**Table 1**). The mean age of the population at the time of biopsy was 61 years (range, 36–86 years). 71% of the samples were from current smokers (n=27), 18% from former smokers (n=7), and 11% from never smokers (n=4). The study included early- and advanced-stage NSCLC patients consisting of 18% (n=7) with stage I, 5% (n=2) with stage II, 24% (n=9) with stage III, and 53% (n=20) with stage IV disease.

**Table 1:** Diagnostic and demographic overview of the NSCLC FFPE clinical cohort.

Patient ID	Cohort	Biopsy Site	Histology	Gender	Age	Stage	Smoking status
Pt. 01	*ALK*	Pleura	ADC	F	75	IV	Former
Pt. 02	*ALK*	Pleura	ADC	M	54	IV	Never
Pt. 03	*ALK*	Pleura	ADC	M	66	IV	Former
Pt. 04	*ALK*	Lymph node	ADC	F	44	IV	Never
Pt. 05	*ALK*	Pleura	ADC	F	73	IV	Former
Pt. 06	*ALK*	Lung	NSCLC NOS	F	38	IV	Former
Pt. 07	*ALK*	Lung	ADC	M	36	IV	Current
Pt. 09 *	*ALK*	Lung	NSCLC NOS	M	63	IV	Former
Pt. 10	*ALK*	Lymph node	ADC	F	86	III	Former
Pt. 11	*ALK*	Bone	ADC	M	65	IV	Current
Pt. 12	*ALK*	Lung	ADC	M	60	IV	Former
Pt. 13	*ALK*	Lung	ADC	F	53	IV	Current
Pt. 14	*MET*	Lung	ADC	M	67	IV	Current
Pt. 15	*MET*	Skin	ADC	M	48	IV	Current
Pt. 16	*MET*	Pericardial fluid	ADC	M	42	IV	Current
Pt. 17	*MET*	Lung	ADC	M	78	II	Current
Pt. 18	*MET*	Lung	ADC	M	80	I	Current
Pt. 19	*MET*	Brain	NSCLC NOS	M	53	III	Current
Pt. 20	*MET*	Lymph node	ADC	M	66	IV	Current
Pt. 21	*MET*	Lung	ADC	M	52	II	Current
Pt. 22	*MET*	Lung	LCC	M	60	III	Current
Pt. 23	*MET*	Lung	ADC	M	40	III	Current
Pt. 24	*MET*	Lung	ADC	M	72	III	Current
Pt. 25	*MET*	Lung	ADC	M	53	III	Current
Pt. 26	*MET*	Lung	ADC	M	64	I	Current
Pt. 27	*MET*	Lymph node	NSCLC NOS	M	73	III	Current
Pt. 28	*MET*	Bone	ADC	M	71	IV	Current
Pt. 29	*MET*	Lung	ADC	M	71	I	Never
Pt. 30	*MET*	Lung	NSCLC NOS	F	45	I	Current
Pt. 31	*MET*	Pleura	ADC	F	81	IV	Never
Pt. 32	*MET*	Lung	ADC	M	52	IV	Current
Pt. 33	*MET*	Lung	ADC	M	51	III	Current
Pt. 34	*MET*	Pleura	ADC	M	65	IV	Current
Pt. 35	*MET*	Lymph node	ADC	F	45	IV	Current
Pt. 36	*MET*	Lung	ADC	F	58	I	Current
Pt. 37	*MET*	Lung	ADC	F	60	I	Current
Pt. 38	*MET*	Lung	ADC	M	73	III	Current
Pt. 39	*MET*	Lung	ADC	M	67	I	Current

Pt., patient; ADC, adenocarcinoma; LCC, large cell carcinoma; NSCLC NOS, non-small cell lung cancer not otherwise specified; F, female; M, male.

*Patient 8 was excluded from the study as a result of a conflict that arose from a contamination issue during sample assessment by the PGDx elio tissue complete assay. Unfortunately, no additional starting material from patient 8 was available for reanalysis.

### *ALK* rearrangements

There was 100% concordance between the PGDx elio tissue complete assay and the orthogonal approaches which included IHC, FISH, and the Oncomine Focus Assay (**Table 2**). *EML4*::*ALK* (E13:A20) was found to be the most prevalent *ALK* fusion comprising 58% of the cases (n=7). Notably, in addition to providing the exact genomic breakpoints for these rearrangements, the PGDx elio tissue complete assay reported the rearrangement types including nine inversions, one duplication within chromosome 2, and two translocations (*IRF2BP2* in chromosome 1 and *KIF5B* in chromosome 10). Notably, the PGDx elio tissue complete identified a novel translocation, *IRF2BP2*::*ALK* (I1:A20), which has not been previously described (**Table 2**; **Supplementary Figure 2**). This sample from Pt. 11 had positive IHC and FISH for the presence of *ALK* rearrangement and an *ALK* imbalance positive expression result when analyzed using the RNA-based NGS Oncomine Focus Assay. The result indicates the presence of a fusion event due to expression imbalance between the 3′ and 5′ ends of the gene, but this NGS approach is not capable of specifically determining the novel gene partner. The DNA-based NGS PGDx elio tissue complete assay allowed the detection of novel fusion genes due to both exonic and intronic probe tiling that captures variants that are not detected through the other methods.

**Table 2:** Detection of *ALK* gene rearrangements in 12 patient cases using IHC, FISH, the Thermo Fisher Oncomine Focus Assay, and the PGDx elio tissue complete assay.

	*ALK* rearrangements								
Patient ID	IHC	FISH		Oncomine Focus Assay		PGDx elio tissue complete			
	Result	Result	Pattern	Result	Fusion details	Result	Gene fusion	Breakpoints	Rearrangement type
Pt. 01	+	+	Split	+	*EML4*::*ALK* (E13:A20)	+	*EML4*::*ALK*	chr2:42525000; chr2:29447000	Inversion
Pt. 04	+	+	Split	+	*EML4*::*ALK* (E13:A20)	+	*EML4*::*ALK*	chr2:42527000; chr2:29447000	Inversion
Pt. 05	+	+	Split	+	*EML4*::*ALK* (E13:A20)	+	*EML4*::*ALK*	chr2:42527000; chr2:29447000	Duplication
Pt. 07	+	+	Split	+	*EML4*::*ALK* (E13:A20)	+	*EML4*::*ALK*	chr2:42525000; chr2:29448000	Inversion
Pt. 12	+	+	Split	+	*EML4*::*ALK* (E13:A20)	+	*EML4*::*ALK*	chr2:42526000; chr2:29448000	Inversion
Pt. 03	+	+	3′ isolated	+	*EML4*::*ALK* (E13:A20)	+	*EML4*::*ALK*	chr2:42524000; chr2:29446000	Inversion
Pt. 10	+	+	3′ isolated	+	*EML4*::*ALK* (E13:A20)	+	*EML4*::*ALK*	chr2:42526000; chr2:29448000	Inversion
Pt. 06	+	+	3′ isolated	+	*EML4*::*ALK* (E6:A20)	+	*EML4*::*ALK*	chr2:42497000; chr2:29447000	Inversion
Pt. 09	+	+	3′ isolated	+	*EML4*::*ALK* (E6:A20)	+	*EML4*::*ALK*	chr2:42496000; chr2:29447000	Inversion
Pt. 13	+	+	Split	N/A	Sample failure	+	*EML4*::*ALK*	chr2:42498000; chr2:29447000	Inversion
Pt. 02	+	+	3′ isolated	+	*KIF5B*::*ALK* (K17:A20)	+	*KIF5B*::*ALK*	chr10:32311000; chr2:29447000	Translocation
Pt. 11	+	+	3′ isolated	+	*ALK* imbalance 0,467	+	*IRF2BP2*::*ALK*	chr1:234745000; chr2:29446000	Translocation

Pt., patient; IHC, immunohistochemistry; FISH, fluorescence *in situ* hybridization; N/A, not available.

**Figure 1:**
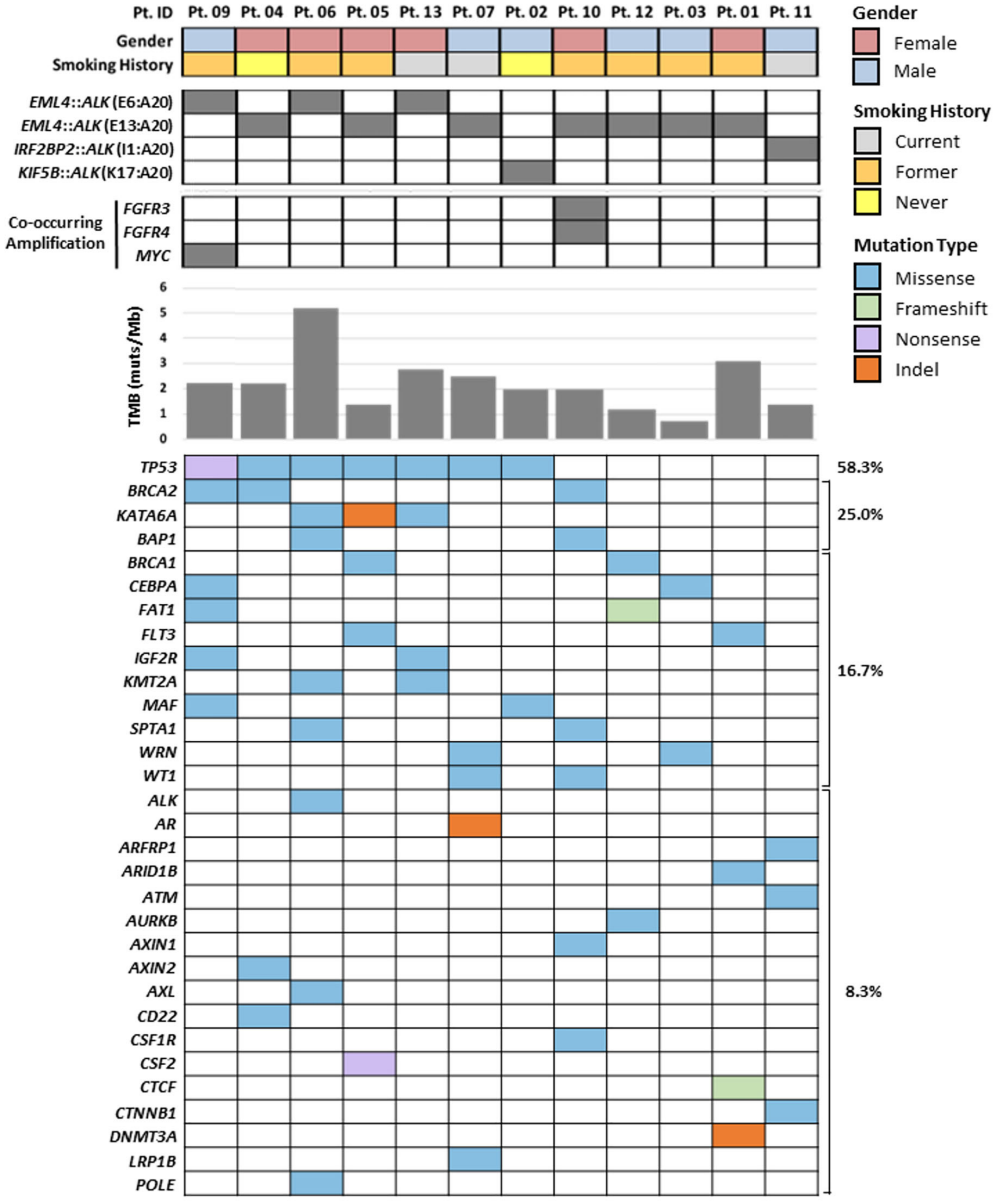
PGDx elio tissue complete comprehensive genomic profiling results of 12 *ALK* fusion-positive patients with demographics correlates. Co-occurring amplifications, TMB scores, and SNVs and indels with the fraction (%) of patient with mutations in each gene are presented.

The concurrent alterations identified by expanded genomic analysis are shown in **Figure 1**. Of the 12 samples processed using the PGDx elio tissue complete, 58.3% were found to harbor concomitant *TP53* alterations, which were found to be predominantly missense mutations. Overall, the majority of *ALK*-rearranged cases were not found to have concomitant gene amplification events, with 83% of cases (n=10) having no gene amplifications present. However, two *ALK*-positive cases were found to have gene amplifications: one with amplifications in both *FGFR3* and *FGFR4* and one with a *MYC* amplification.

### *MET* amplifications

Of the 26 *MET* high GCN samples selected for the PGDx elio tissue complete assay analysis, only eight cases were recategorized as high-amplified (n=2) and medium-amplified (n=6) following the currently accepted FISH criteria. Five out of these eight cases were confirmed as being *MET*-amplified by the PGDx elio tissue complete. Therefore, we found three discordant cases between FISH and NGS amplification calls (**Table 3**). Interestingly, in these cases, increased *MET* copies were detected by the NGS approach but did not meet the PGDx amplification calling criteria, specifically ≥3-fold amplification and at least 25% of the queried regions of *MET* amplified (**Table 3**, numbers denoted in red), and accordingly were not deemed to be genuine amplification events. FISH results from these three cases were reviewed and stated as highly heterogeneous by FISH with focal *MET* amplifications, partially explaining the results observed by NGS, which is based on a bulk tumor analysis (**Supplementary Figure 3**).

**Table 3:** Detection of *MET* amplification in 26 patient cases using FISH and the PGDx elio tissue complete assay.

*MET* amplifications					
Patient ID	FISH		PGDx elio tissue complete		
	Result	*MET* to CEP7 ratio	Result	Copy number	% Regions amplified
Pt. 28	High-amplification	6.1	Amplified	3.2	31.1
Pt. 15	High-amplification	4.1	Amplified	4.5	100
Pt. 16	Medium-amplification	3.2	Amplified	6.6	97.8
Pt. 24	Medium-amplification	2.2	Not amplified	3.3	**4.4**
Pt. 25	Medium-amplification	2.9	Not amplified	3.1	**2.2**
Pt. 26	Medium-amplification	2.4	Not amplified	**2.7**	**20**
Pt. 27	Medium-amplification	2.4	Amplified	3.3	37.8
Pt. 32	Medium-amplification	2.2	Amplified	3.7	35.6
Pt. 14	Low-amplification	1.8	Not amplified	1.0	0
Pt. 17	Low-amplification	1.8	Not amplified	1.0	0
Pt. 18	Low-amplification	1.8	Not amplified	2.1	2.2
Pt. 22	Low-amplification	1.9	Not amplified	1.0	0
Pt. 19	Non-amplification	1.1	Not amplified	1.0	0
Pt. 20	Non-amplification	1.2	Not amplified	1.0	0
Pt. 21	Non-amplification	1.3	Not amplified	1.0	0
Pt. 23	Non-amplification	1.2	Not amplified	2.8	2.2
Pt. 29	Non-amplification	1.0	Not amplified	2.6	37.8
Pt. 30	Non-amplification	1.0	Not amplified	1.0	0
Pt. 31	Non-amplification	1.0	Not amplified	3.3	6.7
Pt. 33	Non-amplification	1.0	Not amplified	1.0	0
Pt. 34	Non-amplification	1.3	Not amplified	1.0	0
Pt. 35	Non-amplification	1.1	Not amplified	3.1	2.2
Pt. 36	Non-amplification	1.3	Not amplified	1.0	0
Pt. 37	Non-amplification	1.2	Not amplified	3.1	6.7
Pt. 38	Non-amplification	1.2	Not amplified	2.6	2.2
Pt. 39	Non-amplification	1.0	Not amplified	4.2	15.6

Numbers denoted in red are in cases where FISH detected *MET* amplification but fell below the amplification calling threshold of the PGDx elio tissue complete assay. Pt., patient; FISH, fluorescence *in situ* hybridization; CEP7, chromosome enumeration probe 7.

**Figure 2:**
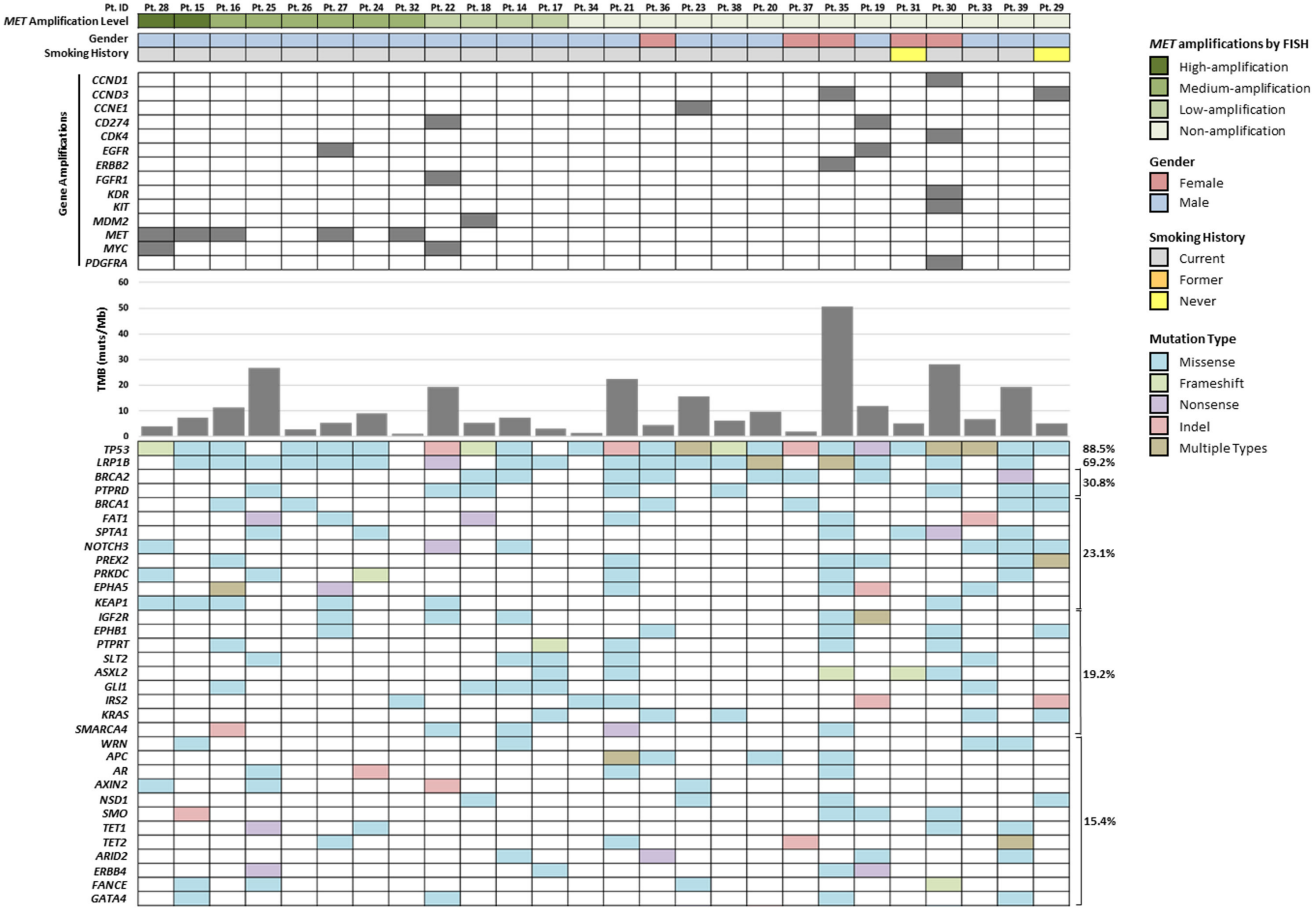
PGDx elio tissue complete comprehensive genomic profiling results of 26 previously characterized *MET* high gene copy number patients with demographic correlates. Amplifications, TMB scores, and SNVs and indels with the fraction (%) of patient with mutations in each gene are presented.

**Figure 3:**
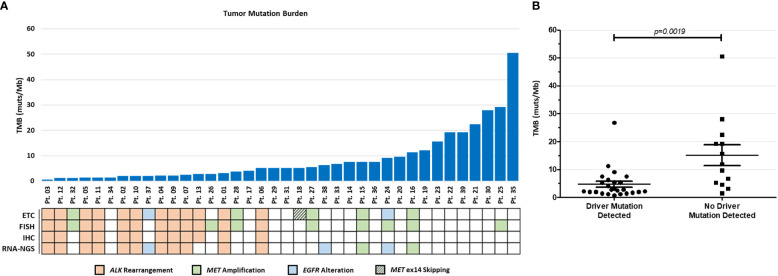
PGDx elio tissue complete TMB results. **(A)** Comparison of TMB scores and canonical driver mutations in all 38 cases. **(B)** A statistically significant inverse relationship between TMB scores and incidence of canonical driver mutations was observed (p = 0.0019).

In a correlative analysis, the five *MET*-amplified cases by both FISH and the PGDx elio tissue complete assay were found not to carry any concurrent targetable driver alterations (Pts. 15, 16, 27, 28, and 32; **Figure 2**). Interestingly, one low-amplified *MET* case by FISH, which was classified as not amplified by the PGDx elio tissue complete assay (Pt. 18), was found to have a concomitant *MET* exon 14 skipping mutation (**Figure 3A**). Five non-amplified cases were found to harbor key NSCLC alternative driver mutations (two *KRAS* exon 2 mutations and three *EGFR* sensitizing mutations) (**Figures 2**, **3A**).

### Genomic signatures

TMB scores in this NSCLC cohort ranged from 0.7 to 50.5 muts/Mb, with a mean TMB of 8.6 and median of 5.2 muts/Mb (**Figure 3A**). Interestingly, there appears to be an inverse relationship between the incidence of canonical NSCLC driver mutations and TMB scores, wherein those cases with identified driver mutations have a significantly lower TMB scores than those with no identified drivers (*p*=0.0019, **Figure 3B**). Regarding microsatellite status, all samples assessed by the PGDx elio tissue complete were found to be microsatellite stable (data not shown).

### Clinical outcomes of cases of interest

Clinical outcome data from specific cases of interest were investigated to assess associations with response to targeted therapy. Patient 11, harboring the newly described *IRF2BP2*::*ALK* translocation, was a 65-year-old man, a 30-pack-year smoker diagnosed with stage IV disease. He received first-line platinum-pemetrexed therapy with progression at first assessment after 2 months. He then initiated crizotinib at standard doses that was efficacious for 4 months when he had central nervous system progression with rapid deterioration, with no further treatment. Two patients with *MET* alterations received anti-MET TKI through clinical trial enrollment. Patient 24 was a 55-year-old man, a 50-pack-year smoker who was classified as *MET* amplified by FISH, although not confirmed by the PGDx elio tissue complete assay. This patient received SAR125844, a selective MET TKI, with stable disease as best response that lasted 4 months. The second was patient 28, classified by FISH as *MET* high GCN and confirmed as amplified by the PGDx elio tissue complete assay. He entered a clinical trial with capmatinib and obtained a partial response that lasted 18 months.

## Discussion

The use of reliable screening methods for detection of molecular alterations in lung cancer is crucial for selecting the appropriate targeted treatment for eligible patients. Our study demonstrates an excellent concordance between all orthogonal methods and the PGDx elio tissue complete NGS assay for *ALK* rearrangements. Although IHC and FISH assays can detect *ALK* fusion events, these methods do not determine *ALK* fusion partners that may predict clinical benefit of selective TKI more accurately (14, 36). Diversity of fusions and how these may affect response to therapies makes comprehensive NGS analysis increasingly necessary for precision oncology.

*EML4*::*ALK* is a canonical structural variant with fusion of exon 13 of *EML4* with exon 20 of *ALK* reported to occur in ~30% of NSCLC cases (37, 38). Interestingly, in studies evaluating the clinical consequence of other *ALK* fusions, many reports have seen no significant difference between *EML4::ALK* and non-*EML4*::*ALK* variants following treatment with targeted ALK inhibitors (39–41). Differences in clinical outcomes, however, have been reported within distinct *EML4*::*ALK* fusion variants, where patients with the canonical *EML4::ALK* (E13:A20) fusion respond better to crizotinib compared with other *EML4*::*ALK* fusion variants (41). More recent studies confirm the prognostic value of different variants, although second- and third-generation ALK inhibitors show better results compared with crizotinib regardless the variant (42). The *IRF2BP2* gene identified as a new translocation partner fused with *ALK* exon 20 codifies for an IFN regulatory factor binding protein that interacts with IFN regulatory factor 2 (IRF‐2). The *IRF2BP2* gene has been described as a fusion partner in acute promyelocytic leukemia when fused to *RARA* gene and in NSCLC is found to be fused to *NTRK1* (43, 44). The patient with the *IRF2BP2*::*ALK* fusion (Pt. 11) received crizotinib as second-line treatment with lower than expected benefit, although progression was at the central nervous system, confirming the limitation of crizotinib at this anatomical site (10). The information provided by comprehensive NGS, evaluated in the context of clinical response to the different ALK inhibitors, may aid in the selection of the optimal treatment strategy for each patient.

In the detection of *MET* copy number status via FISH, the fluorescent signal of *MET* is compared with a CEP7 control and the resultant ratio is reported as a specific level of amplification (19). Through the PGDx elio tissue complete assay, the detected read density for *MET* is compared with the targeted regions overall through a bulk tumor analysis and interpreted bioinformatically in a more quantitative manner. The discrepancies in what constitutes a genuine *MET* amplification arise from the lack of clinically defined and meaningful cutoffs in assessing *MET* amplifications, thereby impeding the adoption of *MET* assessment in standard-of-care workups (15, 16, 45). After approval of MET targeting therapies, a cutoff of 10-fold change detected by NGS has been accepted as the threshold for prescription. However, this remains controversial as assays typically set cutoffs and thresholds for *MET* amplifications respective to their test and system, and therefore, may be problematic as results of *MET* positivity do not translate well across platforms (19, 46).

In our series, of 26 *MET* high GCN cases reclassified according to current FISH criteria, (19) only eight were considered *MET* amplified (two high-amplified and six medium-amplified). Analyzing by NGS with the PGDx elio tissue complete assay, we observed that five of these eight were also considered amplified, with only three discordant cases (discussed later). The other 22 *MET* high GCN cases analyzed in this study were divided by current FISH criteria into 4 low-amplified and 15 non-amplified. The agreement with the NGS panel in these 22 cases was 100% since *MET* amplification was not detected in any of them. Even so, the copy number values resulting from NGS of Pt. 29 and Pt. 39 stand out, with a *MET* copy number adjusted to the tumor of 2.6% and 37.8% of regions amplified, and Pt. 39, with a *MET* copy number of 4.2% and 15.6% of regions amplified, respectively. Both cases displayed copy number variation but did not reach the calling threshold to indicate an amplification by the PGDx elio tissue complete assay (≥3-fold copy number in at least 25% of the queried regions), which is consistent with heterogeneous copy number events such as *MET* gene gains or chromosome 7 polysomy (47–49). As opposed to gains or polysomy, amplification is thought to represent a state of true biologic selection for MET activation as an oncogenic driver (16). In published results of clinical trials in response to MET inhibitors, clinical benefit was only observed in patients with the so-called “true” amplification (18, 19, 50).

Consistent with this, the prevalence rate of *MET* amplifications in NSCLC can range from 1% to 5%, with estimates varying depending on the assay used and the fold amplification thresholds implemented. Notably, *MET*-amplified NSCLC patients have been observed to benefit from certain targeted inhibitors, such as crizotinib; however, the magnitude of benefit is below what is observed with other driver alterations, highlighting the relevance of defining and harmonizing the cutoff (51–55). Interestingly, from the two patients treated with MET inhibitors in our series, the one with the discordant results with PGDx elio tissue complete showed a poor (4-month) performance of TKI treatment compared with the patient with confirmed *MET* amplification by both techniques.

Whereas detecting rearrangement events via FISH and NGS shows high concordance, detecting copy numbers for genomic amplifications remains more ambiguous. Recent studies are addressing this clinical gap. In the study of capmatinib, patients whose tumors harbored *MET* GCN of at least 10 exhibited greater therapeutic benefit; however, the response rates were relatively low (40% for treatment naïve vs. 29% for previously treated) compared with patients with exon 14 skipping mutations in *MET* in the same scenarios (18). In that study, *MET* GCN alterations were defined by FISH and retrospectively assessed by NGS using the FoundationOne CDx panel. No correlation of both assessments has been reported to our knowledge. In another study with tepotinib, *MET* amplification assessed by Guardant 360^®^ technology with a cutoff of CN ≥2.5 in liquid biopsy in the absence of *MET* mutations, tepotinib showed encouraging results (ORR 71% in treatment naïve vs. 30% in second- and third-line settings) (25). These results might better reflect a more biologically relevant cutoff defined by NGS in this study.

Regarding the inverse relationship between TMB score and the presence of canonical driver alterations, it has been previously described that *EGFR* mutant lung cancers featured lower overall TMB as compared with *EGFR* wild‐type cancers (56). Also, potential clinical implications have been seen between *EGFR*-positive patients with low/high TMB when treated with TKIs (57). One limitation of our study is that we do not have sufficient statistical power to compare the parameters of response to treatment and overall survival because both *ALK*-fused and *MET*-amplified cases were pooled together in the driver alteration-detected group. However, this inverse association between high TMB and the presence of an *ALK* fusion was also observed previously, pointing out its potential interest when designing a precision therapy strategy for these patients (58, 59).

The discordant *MET* results observed in our study might derive from different technical aspects. The PGDx elio tissue complete assay considers factors such as tumor purity, percent of the sequenced genomic region amplified, and minimum coverage depths in determining whether the observed increase in copy number constitutes a genuine amplification event. Such metrics are not taken into consideration with FISH, instead relying on the subjectivity of the observer when deciding on which areas to assess in identifying copy number changes. Bias generated by FISH count due to tissue heterogeneity could potentially differ between tissue sections as reported to occur in the context of other biomarkers and indication, for example, HER2 assessment in breast cancer (60). Despite this, NGS approaches do not solve this biological phenomenon and *MET* focal amplifications could require further assessment by FISH, as these might not be captured by NGS if the majority of cells do not harbor the amplification. There is no specific information of clinical responses in these particular cases.

## Conclusions

The PGDx elio tissue complete assay demonstrated high overall concordance with conventional diagnostic approaches for *ALK* gene rearrangements. Additionally, the DNA-based NGS approach provides far greater insight into the underlying genomic events with the ability to identify exact breakpoints of gene fusions and report the specific loci associated with gene amplifications. Moreover, it provides broader information regarding potentially relevant genomic co-alterations that may impact clinical outcomes. The optimal cutoff point for defining *MET* amplifications remains to be defined and might be specific to the NGS platform. Further studies with clinical outcome data are needed to further refine *MET* amplification as a predictive biomarker.

## Data availability statement

The original contributions presented in the study are publicly available. This data can be found here: https://ega-archive.org/datasets/EGAD50000000016. Accession number - EGA50000000010.

## Ethics statement

Ethical review and approval was not required for the study on human participants in accordance with the local legislation and institutional requirements. Written informed consent from the patients/participants or patients/participants’ legal guardian/next of kin was not required to participate in this study in accordance with the national legislation and the institutional requirements.

## Author contributions

SC, JJ, MS, EK, EW, DN, JS, BB, and EA contributed to conception and design of the study. JJ, JK, KG, EV, EK, EW, JH, GC, DN, and JS organized the database. JJ, EK, and JG performed the statistical analysis. SC and EK wrote the first draft of the manuscript. JJ, MS, JK, KG, EV, LP, BB, and EA wrote sections of the manuscript. MH-W, PR, XR, and ET participate in image acquisition and technical support in immunohistochemistry, fluorescence *in situ* hybridization and next-generation sequencing. All authors contributed to the article and approved the submitted version.

## Supplementary material

Supplementary Figure 1 Representative H&E and FISH images of cases illustrating the currently accepted FISH criteria for *MET* copy number classification. Samples considered with high *MET* GCN were reclassified according to the current FISH-criteria as **(A)** high-amplification (*MET*/CEP7 ratio ≥4.0), **(B)** medium-amplification (*MET*/CEP7 ratio >2.2-<4.0), and **(C)** low-amplification (*MET*/CEP7 ratio ≥1.8-≤2.2).

Supplementary Figure 2 Representative microscopy images of Patient 11, an *ALK*-positive case with the *IRF2BP2*::*ALK* (I1:A20) novel fusion partner. **(A)** Patient was a 65-year-old man diagnosed of a stage IV lung adenocarcinoma showing an acinar predominant pattern with signed ring cells (10X). **(B)** ALK D5F3 (Ventana) positive expression (20X). **(C)** FISH positive pattern showing isolated 3’*ALK* signals (100X).

Supplementary Figure 3 Representative H&E and FISH images of one *MET* discordant case (Patient 26) between FISH and NGS. **(A)** Non-amplified case with chromosome 7 polysomy and classified as *MET* high copy number gain according to Cappuzzo score (*MET* GCN ≥5.0). Case presented a highly heterogeneous FISH counting with areas with **(B)** focal *MET* amplifications with a high CEP7 enumeration and areas **(C)** without *MET* amplification and presenting chromosome 7 polysomy.

Supplementary Table 1 Quality control parameters for NGS using the PGDx elio tissue complete assay.
