# Supplementary figures and images for: Comprehensive NGS profiling to enable detection of ALK gene rearrangements and MET amplifications in non-small cell lung cancer
Source: Front Oncol. 2023 Oct 20;13:1225646. doi: 10.3389/fonc.2023.1225646 (PMC10623306; doi:10.3389/fonc.2023.1225646)

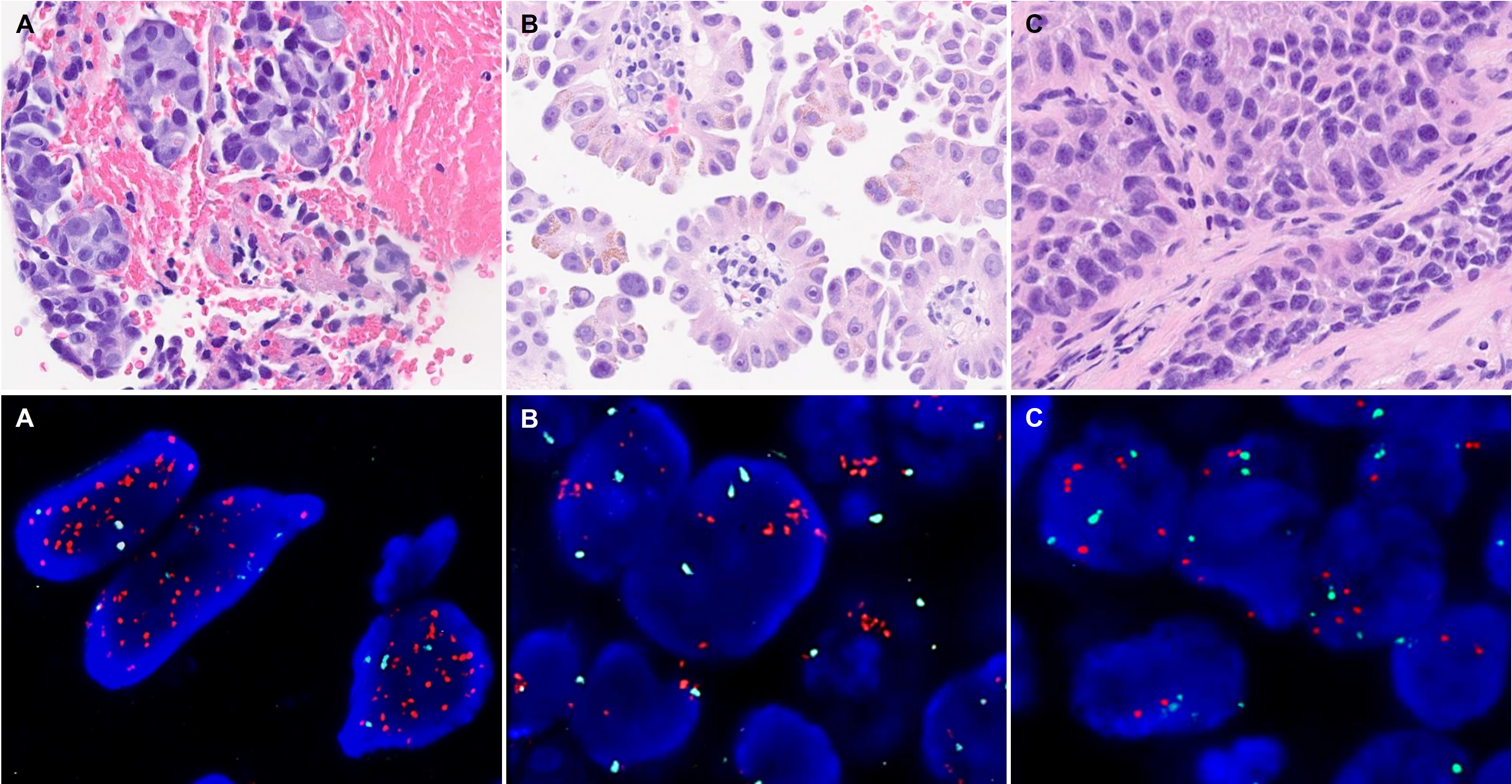

Supplement: Supplementary Figure 1 — Representative H&E and FISH images of cases illustrating the currently accepted FISH criteria for MET copy number classification. Samples considered with high MET GCN were reclassified according to the current FISH-criteria as (A) high-amplification (MET/CEP7 ratio ≥4.0), (B) medium-amplification (MET/CEP7 ratio >2.2-<4.0), and (C) low-amplification (MET/CEP7 ratio ≥1.8-≤2.2). [file Image_1.jpeg]

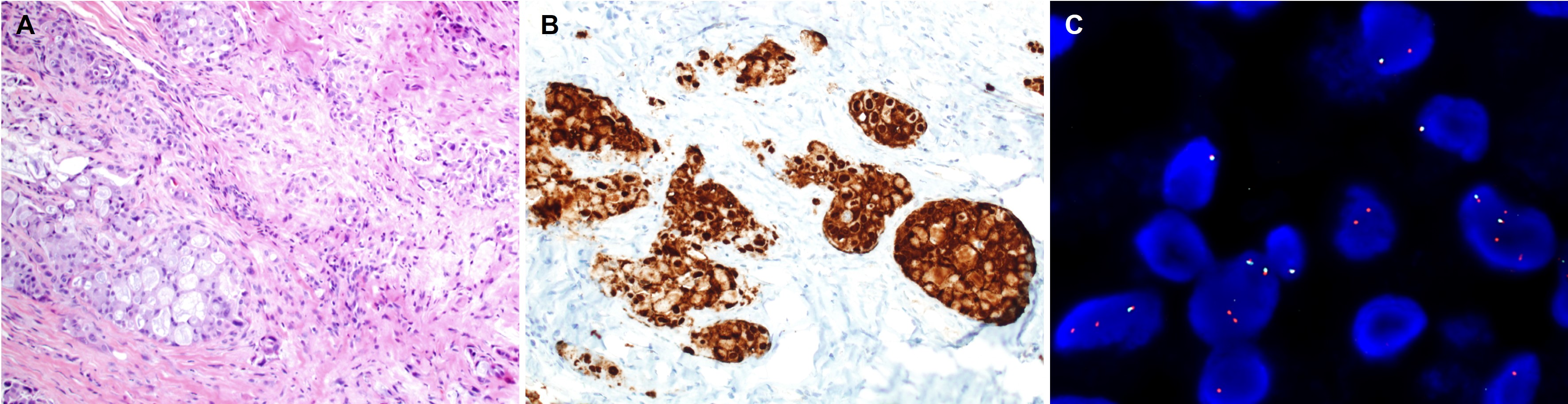

Supplement: Supplementary Figure 2 — Representative microscopy images of Patient 11, an ALK-positive case with the IRF2BP2::ALK (I1:A20) novel fusion partner. (A) Patient was a 65-year-old man diagnosed of a stage IV lung adenocarcinoma showing an acinar predominant pattern with signed ring cells (10X). (B) ALK D5F3 (Ventana) positive expression (20X). (C) FISH positive pattern showing isolated 3’ALK signals (100X). [file Image_2.jpeg]

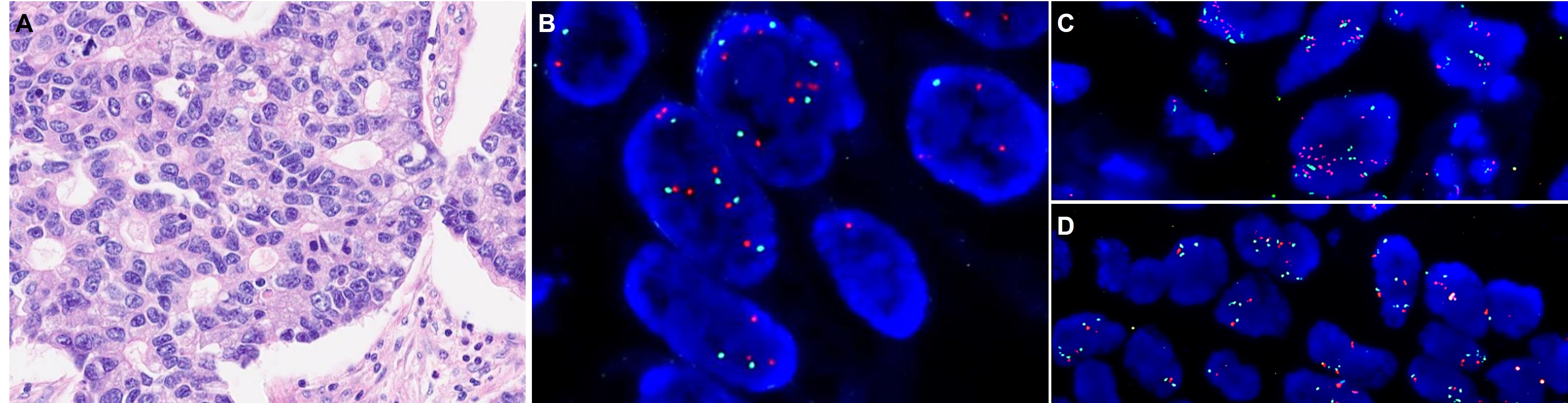

Supplement: Supplementary Figure 3 — Representative H&E and FISH images of one MET discordant case (Patient 26) between FISH and NGS. (A) Non-amplified case with chromosome 7 polysomy and classified as MET high copy number gain according to Cappuzzo score (MET GCN ≥5.0). Case presented a highly heterogeneous FISH counting with areas with (B) focal MET amplifications with a high CEP7 enumeration and areas (C) without MET amplification and presenting chromosome 7 polysomy. [file Image_3.jpeg]
