# Supplementary material for: Comprehensive NGS profiling to enable detection of ALK gene rearrangements and MET amplifications in non-small cell lung cancer
Source: Front Oncol. 2023 Oct 20;13:1225646. doi: 10.3389/fonc.2023.1225646 (PMC10623306; doi:10.3389/fonc.2023.1225646)
Supplement: Supplementary Table 1 — Quality control parameters for NGS using the PGDx elio tissue complete assay. [file Table_1.docx]

**Supplemental Table**

| **Quality Control Metric** | **PGDx elio tissue complete RUO NGS assay** |
| --- | --- |
| DNA Input | 100 ng (50 ng minimum) |
| Minimum Tumor Purity | 20% Tumor Content |
| Library QC | ≥ 15 ng/uL within 180-800 bp; minimum average length 250 bp |
| Capture QC | ≥ 10 nM within 180-800 bp; minimum average length 250 bp; dimers ≤ 5% |
| Sequencing QC | ≥ 130 cluster density; Q30 R1/R4 of 80% and Q30 R2/R3 of ≥ 85% within 15-35% |
| Targeted Bases | ≥ 90% of targeted regions with a minimum >100x coverage |
| Contamination QC | Sample must pass SNP analysis |
| Control QC | Sample must have a passing control |

**Supplemental Table 1.** Quality control parameters for NGS using the PGDx elio tissue complete assay.

Abbreviations: RUO (Research use only); QC (Quality control); Q30 (Quality score of 30); SNP (Single nucleotide polymorphism).
